# Stable Isotope Abundance and Fractionation in Human Diseases

**DOI:** 10.3390/metabo11060370

**Published:** 2021-06-09

**Authors:** Illa Tea, Arnaud De Luca, Anne-Marie Schiphorst, Mathilde Grand, Sophie Barillé-Nion, Eric Mirallié, Delphine Drui, Michel Krempf, Régis Hankard, Guillaume Tcherkez

**Affiliations:** 1 Université de Nantes, CNRS, CEISAM UMR 6230, 44000 Nantes, France; Anne-Marie.Schiphorst@univ-nantes.fr (A.-M.S.); Mathilde.Grand@univ-nantes.fr (M.G.); 2 Research School of Biology, ANU College of Science, Australian National University, Canberra, ACT 2601, Australia; guillaume.tcherkez@anu.edu.au; 3 Inserm UMR1069 «Nutrition, Croissance et Cancer», bât Dutrochet, 10 bd Tonnellé, CEDEX, 37032 Tours, France; arnaud.deluca@univ-tours.fr (A.D.L.); regis.hankard@inserm.fr (R.H.); 4 Université de Tours, 10 bd Tonnellé, CEDEX, 37032 Tours, France; 5 Université de Nantes, Inserm UMR 1232, CRCINA, 44000 Nantes, France; sophie.barille@univ-nantes.fr; 6 Université de Nantes, Chirurgie Cancérologie Digestive et Endocrinienne, Institut des Maladies de l’Appareil Digestif, Hôtel Dieu, CHU Nantes, Place Ricordeau, CEDEX 1, 44093 Nantes, France; Eric.Mirallie@univ-nantes.fr; 7 Service Endocrinologie, Diabétologie, Nutrition, CHU de Nantes, Bd J. Monod. Saint Herblain, CEDEX 1, 44093 Nantes, France; Delphine.DRUI@chu-nantes.fr; 8 Université de Nantes, Elsan Clinique Breteché, 44000 Nantes, France; Michel.KREMPF@clinique-breteche.fr; 9 Institut de Recherche en Horticulture et Semences, INRAe, Université d’Angers, 49070 Beaucouzé, France

**Keywords:** isotope effect, fractionation, metabolic partitioning, diabetes, cancer, metal homeostasis

## Abstract

The natural abundance of heavy stable isotopes (^13^C, ^15^N, ^18^O, etc.) is now of considerable importance in many research fields, including human physiology. In fact, it varies between tissues and metabolites due to isotope effects in biological processes, that is, isotope discriminations between heavy and light isotopic forms during enzyme or transporter activity. The metabolic deregulation associated with many diseases leads to alterations in metabolic fluxes, resulting in changes in isotope abundance that can be identified easily with current isotope ratio technologies. In this review, we summarize the current knowledge on changes in natural isotope composition in samples (including various tissues, hair, plasma, saliva) found in patients compared to controls, caused by human diseases. We discuss the metabolic origin of such isotope fractionations and highlight the potential of using isotopes at natural abundance for medical diagnosis and/or prognostic.

## 1. Introduction

It is now more than a century that Marie Curie Skłodowska provided key advances on (radioactive) isotopes and how they can be used for human health. In the second part of the XXth century, stable isotopes have then been exploited in medicine and biomedical research, mostly using enriched material (isotopic labelling with heavy water ^2^H_2_O, ^13^C-enriched glucose, leucine or urea, …) to quantify water turn-over, follow blood glucose homeostasis or trace the fate of precursors in metabolic pathways (for reviews, see [1,2,3]). Intense efforts are currently devoted to set up diagnostic procedures for metabolism-based pathologies using isotopic labelling. As an example, ^13^C-phenylalanine and subsequent measurement of respired ^13^CO_2_ has recently been proposed for the diagnosis of phenylketonuria, a well-known inborn metabolic disease [4]. The use of isotopically enriched material has two drawbacks: first, it is rather expensive and second, feeding or injecting isotopic products may be associated with long procedures to address ethical or safety imperatives (e.g., European regulation no. 536/2014), although the safety of using stable isotopes is well established [5,6].

During the past two decades, key advances have been made in our knowledge of natural isotope abundance (i.e., without isotopic enrichment) to take advantage of small but detectable differences in natural isotope content between patients and controls, associated with quite a range of pathologies. In fact, all of the natural elements are present in the form of various isotopic forms (e.g., ^12^C and ^13^C for carbon) and some changes in isotope ratios (i.e., fractionations) have been found to be specific of diseases, reflecting key alterations in metabolism. In this mini-review, we summarize the current knowledge in fractionations associated with human diseases and discuss the potential of using isotopes at natural abundance for medical diagnosis and/or prognostic.

## 2. Basics of Stable Isotopes and Metabolic Isotope Effects

### 2.1. General Principles

Elements forming biological tissues have different stable isotopes, like carbon (^12^C and ^13^C) and nitrogen (^14^N and ^15^N) for which the heavy form represents about 1.1 and 0.37%, respectively. However, this percentage varies amongst organs, tissues and metabolites and also between C and N atom positions within metabolites. For example, lipids are ^13^C-depleted compared to sugars, body proteins are generally ^15^N-enriched compared to exogenous diet proteins, etc. These differences are mostly due to isotope effects, whereby the velocity of enzymatic reactions or transport phenomenon differ between isotopic forms (in the Appendix A, Box A1 for definitions). It is already well-known that decarboxylation reactions discriminate between ^12^C and ^13^C so that evolved CO_2_ is generally ^13^C-depleted compared to the substrate, and deamination reactions fractionate against ^15^N so that amino acids left behind are ^15^N-enriched (and thus excreted ammonia is ^15^N-depleted). The isotope discrimination (or fractionation), denoted as Δ, is often quantified using the difference between substrate and product isotope composition (or delta value, denoted as δ^13^C, δ^15^N, etc.), i.e., Δ = δ_substrate_ − δ_product_. The term “isotope composition” refers to the isotope ratio relative to the international standard (usually measured with isotope ratio mass spectrometry, in the Appendix A, Box A2). Thus, delta values are negative (positive) when the sample of interest contains less (more) heavy isotope than the international standard. Both delta values and fractionations are small numbers and are expressed in per mil (‰).

The fact that isotope effects arise from enzyme action or transport explains why changes in metabolic pathways often lead to changes in delta values. Alterations in delta values can also stem from a source effect, whereby the origin and thus the delta value of the substrate has changed. A well-known example of source effect is the natural isotopic difference between C_3_ (wheat, rice, beet, etc.) and C_4_ plants (maize, sorghum, sugar cane, etc.), the latter being naturally ^13^C-enriched by up to 20‰ compared to the former. This difference allows facile tracing of C_4_ plants consumption and thus potentially, identification of maize utilisation in food products. A recent interesting example involving both source and metabolic effects has been published recently using a giant type I bladder stone (whewellite) from a Chinese 70 y-old patient. The bladder stone has been analysed layer after layer, and the δ^13^C value has been found to correlate positively to calcium oxalate content while the δ^15^N value correlated negatively to struvite (magnesium ammonium phosphate, MgNH_4_PO_4_∙6 H_2_O) content [7]. This indicates that (i) the δ^13^C of excreted oxalate was very high (near −10‰) pointing to a plant origin (oxalic-rich food such as strong black tea and low-value vegetables), and (ii) the δ^15^N of ammonia was relatively depleted (near +4‰) reflecting the isotope effect in amino acid deamination reactions.

Medical applications of natural abundance to detect pathologies are based on these principles, that is, a change in delta values caused by alterations in metabolism, nutritional conditions, or recycling efficiency (e.g., hepatic remobilisation). There has been an exponential increase in studies looking at potential changes in delta values in hair, blood (serum or clot), or other sample types associated with a large range of diseases, from metabolic disorders to cancer (summarized in Table 1). It should be kept in mind, however, that resolving the influence of confounding factors can remain challenging. First, there are important differences in diet composition between countries and therefore, delta values are not always comparable (for a recent survey, see [8]). This implies that it is preferable to use relative (i.e., isotopic offset with respect to food intake delta values) rather than absolute delta values. Second, cohorts must be formed with care since there can be unforeseen isotopic differences caused by common medical treatments, local nutritional habits and also importantly, sex [9].

### 2.2. Isotopes in Metabolism Preclinical Studies

There is now considerable evidence that pathologies directly related to metabolism (nutritional stress, malnutrition, metabolic syndrome in general, diabetes, and obesity) have an impact on natural isotope abundance. Pre-clinical studies with rats subjected to caloric restriction, normal or high fat diet regimes have demonstrated that caloric restriction causes a general decline in peripheric protein content but the impact on isotope compositions varies between organs [36]. In fact, compared to food intake, liver and plasma proteins are ^15^N-enriched under caloric restriction while muscles (heart and skeletal muscles) turn to be ^15^N-depleted [37,38]. In addition, the δ^13^C value in proteins decreases in all tissues, showing a general ^13^C-depletion. Such variations are due to changes in amino acid homeostasis, whereby liver oxidises more amino acids and this process discriminates between isotopes (against ^15^N). Therefore, amino acids left behind and available for protein synthesis are ^15^N-enriched. Also, the commitment of ingested carbohydrates to biosynthesis of non-essential amino acids increases (at the expense of catabolism and respiration) and as a result, proteins are ^13^C-depleted.

### 2.3. Isotopes in Human Metabolic Syndrome, Diabetes, or Nutritional Stress

In humans, several studies took advantage of the delta value of easily accessible samples (blood, hairs) in prediabetic patients or in association with physiological variables. A typical situation has been found in patients affected by nervosa anorexia and nutritional stress during pregnancy, whereby the δ^15^N values in hair increases, showing the involvement of recycling leading to ^15^N-enriched amino acids [10,11]. Conversely, an increase in body mass index is associated with an increase in δ^13^C in hair showing the utilisation of diet proteins [10]. The relationship between isotope abundance in hair and nutritional stress has been reviewed elsewhere [39]. Interestingly, natural isotope abundances are also clearly affected by malnutrition. In collagen extracted from tibiae bone powder (from early XXth century skeletons), syphilitic patients have been found to have a δ^15^N value up to 0.4‰ lower than controls, while no difference in δ^13^C was found [12]. It is probable that this effect is caused by malnutrition induced by either syphilis itself (inflammation of the digestive tract) but also from the treatment used in the past (mercury) which causes intestinal lesions and appetite loss. Similarly, in children from Bangladesh with chronic malnutrition and potential growth retardation (stunted children), hairs are both ^13^C- and ^15^N-depleted [13]. This effect probably reflects a poor-diet effect whereby low-value food is consumed by either children after weaning, or mothers during pregnancy and lactation.

Hair δ^15^N has also been shown to increase in patients with metabolic syndrome, while the δ^13^C value decreases [14]. However, quantitative relationships (regressions) are only significant with glycaemic index and waist circumference. Also, the δ^13^C value is lower when glycaemia increases, or plasmatic high density lipoprotein (HDL) cholesterol concentration is low. Although this study suggests a link between isotope abundance and remobilisation (amino acid and protein recycling) or sugar utilisation, potential confounding factors were recognized (age, nutritional habits). In a comparison of diabetic patients with controls, no change in hair δ^15^N was found while the δ^13^C value declined and a relationship was found with haemoglobin A1 glycation (HbA1c), this relationship being mostly visible in males [15]. However, in another cohort, hair δ^15^N correlated to plasmatic leptin concentration (in particular in individuals with high body mass index) but no relationship was found with δ^13^C values [16]. In adolescents or children, the δ^13^C value in fingerstick blood samples or in erythrocytes has been found to increase at high food intake (increased sugar intake or high calory diet) and this seemed to be unrelated to HbA1c or to C_4_ sugar consumption [17,40]. Also, no difference in hair δ^15^N between type 2 diabetes mellitus patients with or without diabetic nephropathy has been found, although hair δ^15^N correlates to estimated glomerular filtration rate (eGFR) [41].

Since amino acid metabolism (via transamination) is very active in the liver and is associated with different isotope effects [42], changes in δ^15^N values (and potentially, δ^13^C values as well) can be anticipated in metabolic diseases affecting liver function, including in cirrhotic patients. Petzke et al. [19] compared the δ^15^N and δ^13^C values of hair from patients with liver disease to healthy controls. Bulk protein δ^15^N was 3.2‰ lower in cirrhotic patients compared to controls without any significant differences in δ^13^C. Additionally, nearly all amino acids had a lower δ^15^N in cirrhotic individuals. This effect was probably due to a “decreased nitrogen disposal”, that is, an impairment of N remobilization and thus retention of nitrogen that is normally used and excreted in healthy individuals [34].

### 2.4. Metabolic Diseases and Isotope Composition of Respired CO_2_

As discussed above, alterations in isotope compositions with metabolic diseases have been found, but there are presently some undesirable variations across studies. Of course, part of these variations comes from the degree of insulin resistance, glucose capture by muscles and glucose output by the liver, as well as the inflammatory response of the adipose tissue (lipotoxicity). Such physio-pathological factors of obesity and metabolic syndrome, which vary between patients, probably contribute to isotopic variations. Noteworthy, isotopic variations also likely come from differences in country-specific food intake behaviour and thus how patients with diabetes and metabolic syndrome readjust their diet. Still, there is no specific δ^13^C signature of exhaled breath CO_2_ in (pre)diabetic patients [18,43]. It has been suggested that (pre)diabetic patients have an altered carbonic anhydrase activity, causing a change in CO_2_-water oxygen equilibration in tissues and thus a specific δ^18^O value in exhaled CO_2_ [18]. This remains controversial since no highly significant difference was found in another study [43]. An interesting study has nevertheless shown a link between respired CO_2_ and systemic inflammation [32]. In effect, δ^13^C values of exhaled breath CO_2_ in ventilated paediatric patients (infants) in intensive care unit were not different between groups with systemic inflammatory response syndrome (SRIS), no SIRS and SIRS with shock; however, breath δ^13^C value was significantly lower in patients with active sepsis (septic shock), trauma, or after surgery compared to other individuals. Thus, breath δ^13^C does not appear to be related to SIRS but rather, to the occurrence or severity of systemic inflammation [44].

## 3. Isotope Fractionation in Cancer

Over the past two decades, isotopes of both macro-elements (C, N, S) and metals have been investigated in biological samples from patients with cancer. In this section, we shall focus on macro-elements so as to relate to potential alterations in cancer metabolism. Metal stable isotopes in cancer are addressed in the next section.

### 3.1. Cancer Cell Metabolism: Why Might Isotopes Be Impacted?

**Figure 1:**
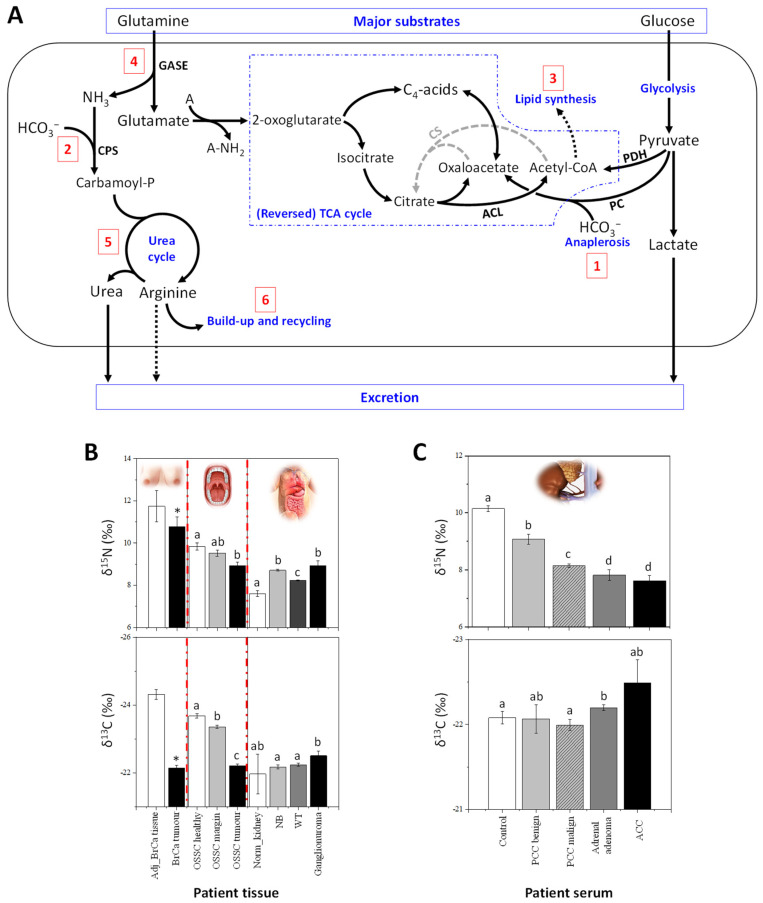
Natural ^13^C and ^15^N abundance in cancerous tissues. (**A**) Major metabolic pathways explaining the isotope abundance in cancerous cells (redrawn from [20]): the ^13^C-enrichment mostly comes from the anaplerotic fixation of bicarbonate by pyruvate carboxylase (PC, 1) and carbamoyl-phosphate synthase (CPS, 2) to feed the urea cycle, as well as a lower accumulation and δ^13^C value of non-structural lipids (3); the ^15^N-depletion comes from the consumption of glutamine via glutaminase (GASE, 4), isotope effects in the urea cycle (5) and a decreased excretion of ^15^N-depleted arginine (6). Abbreviations: A, generic amino group acceptor; CS, citrate synthase; PDH, pyruvate dehydrogenase complex. (**B**) Changes in δ^15^N and δ^13^C in breast cancer (BrCa) tissues, oral squamous cell carcinomas (OSCC) [22] and infant cancer patients [23,24,25]. δ^15^N and δ^13^C can differentiate adjacent non-cancerous BrCa tissue (Adj BrCa) and tumour tissue (BrCa tumour) [20]. Also, δ^15^N and δ^13^C values in OSCC tumour tissues (OSCC tumour) slightly differ from tissues from margin (OSCC margin) and distant oral mucosa (OSCC healthy) [22]. In babies or children, δ^15^N and δ^13^C values from ganglioneuroma (benign tumours), neuroblastoma (NB), and nephroblastoma (Wilm’s tumours, WT, which are malignant) are compared to normal kidney cortex tissue (Norm_kydney), used as a control [23,24]. (**C**) In adults, changes in δ^15^N and δ^13^C in serum of patients with different types of adrenal gland cancers: pheochromocytoma (PCC malignant, *n* = 7; PCC benign, *n* = 6), adrenal adenoma (*n* = 10) and adrenocortical carcinoma (ACC, *n* = 4) which are compared to healthy patients (*n* = 23) used as controls (unpublished data). Letters above bars stand for statistical classes (ANOVA, *p* < 0.05). The asterisk indicates statistical significance in pairwise comparison (*p* < 0.05).

Presumably, metabolism deregulation in cancer should lead to strong alterations in the abundances of natural isotopes ^13^C, ^15^N and ^34^S since many metabolic pathways accompany oncogenesis. In particular, cancer cells adapt their metabolism to maximize the use of N and C sources for anabolism and biosynthesis of macromolecules required by cell proliferation and tumour growth [45]. It is now well established that cancerous cells show increased glucose and glutamine consumption rates, leading to lactate production as well as augmented nitrogen excretion. In particular, urea cycle (UC) deregulation (recently reviewed in [45]) must in principle result in variations in δ^13^C and δ^15^N values (summarized in Figure 1A). How delta values vary in cancerous cells and tissues was unknown until the first investigation was released in a patent based on EA-IRMS technology (see Appendix A, Box A2) applied to biological fluids or tissues associated with cancer [21]. In what follows, we start with breast cancer (BrCa), which is presently the best documented cancer type as isotopes are concerned.

### 3.2. Breast Cancer

δ^13^C and δ^15^N values have been measured on a set of both exeresis samples from patients and cultured BrCa cell lines, showing that cancerous cells with propensity to be invasive are naturally ^15^N depleted and ^13^C enriched (Figure 1B) [20]. Furthermore, by using compound-specific analyses (see Box A2), it has been demonstrated that the generation of ^15^N-depleted arginine and urea by the UC is likely to be at the origin of the ^15^N depletion in cancerous cells. Also, the natural ^13^C-enrichment is probably due to anaplerotic C fixation (organic acids formed by pyruvate carboxylase) and/or decreased accumulation of lipids (which are naturally ^13^C-depleted). In addition, the UC incorporates bicarbonate (naturally ^13^C enriched) via carbamoyl phosphate and thus, the arginine build-up contributes to the natural ^13^C enrichment in cancerous cells (summarized in Figure 1A). It is worth noting that delta values have a good potential to distinguishing BrCa subtypes, since they have been shown to have different metabolic phenotypes [46]. For example, cholesterol biosynthesis is stimulated in triple negative BrCa lines [47], meaning that the δ^13^C cholesterol might change.

### 3.3. Oral Squamous Cell Carcinomas

Other cancers also appear to be associated with alterations in delta values consistent with BrCa. Oral tissue δ^15^N has been shown to decrease in patients with oral squamous cell carcinomas (OSCC), while δ ^13^C values increase [22]. Also, δ^15^N and δ^13^C values slightly differ between tissues taken from margin and distant oral mucosa (Figure 1B). Interestingly, this study also revealed that tumour δ^13^C is positively correlated with alcohol consumption and occurrence of angioinvasion, and inversely related with BMI index.

### 3.4. Cancer in Infants

In infants or children, the δ^15^N values in tissues from ganglioneuroma (benign tumours), neuroblastoma and nephroblastoma (Wilm’s tumours, WT, which are malignant) have been found to be higher compared to normal kidney cortex tissue, used as a control [23,24] (summarized in Figure 1B). Isotopic signatures of WT in subsequent stages of cancer disease have also been studied and WT tissues were ^15^N-depleted and ^13^C-enriched in stage 3 of the disease compared to stage 2. In a comparison of blastemal WT with anaplasia (focal type), it has been shown that the δ^15^N values of blastemal WT declined and seemed to be related to a high protein synthesis rate, which characterizes tumours with features of anaplasia and result in aggressive (and usually fatal) cases [24]. Anaplasia is an independent prognostic factor in WT, according to the definition introduced by the National Wilms’ Tumour Study and subsequent modifications [48,49]. It is considered to be a negative prognostic marker when it appears as a diffuse feature or when it is recognized in an advanced stage of the disease; in such situations, treatment intensification is necessary [50,51,52], due to the greater resistance of the tumour to chemotherapy [52,53]. In this context, low δ^15^N values might be good biomarkers of the worst prognosis [24]. Amongst childhood tumours, rhabdomyosarcoma (RMS) is relatively uncommon, with two major histological types, embryonal (ERMS) and alveolar (ARMS). Five-year survival is observed in approximately 82% for ERMS and 65% for ARMS [25], and the prognosis for patients with progressive disease is still poor. Preliminary results have shown that ARMS leads to ^15^N-depleted muscles tissues relative to ERMS, with no change in δ^13^C [25].

### 3.5. Adrenal Gland Cancer

In adults, ^15^N-depletion in serum was also found in different types of adrenal gland cancers (Figure 1C) compared to healthy patients (unpublished data from our laboratory). Adrenal gland cancers are rare diseases and adrenocortical carcinoma (ACC) is characterized by strong malignancy. The serum of patients with contrasting types of adrenal tumors (i.e., ACC, adrenal adenoma and PPC, pheochromocytoma) has been analysed and low δ^15^N values significantly correlate to malignancy (Figure 1C). For example, patients with ACC have a naturally ^15^N-depleted serum compared to patient with PCC or adrenal adenomas. Moreover, in the group of patients with PCC, patients with benign tumours can be differentiated from those with malignant tumours on a δ^15^N basis. 

This suggests a strong impact of malignancy on PCC cancer cell metabolism, which in turn affects δ^15^N values in circulating metabolites. By contrast, no change in δ^13^C values was observed. Although the metabolism of the different types of adrenal gland cancer is not well-known, an interesting feature is that the glucose transporter (GLUT1) is a promising prognostic marker of ACC [54]. Since glucose entry and insulin-based regulation is essential for protein turn-over signalling and amino acid cellular homeostasis, it is possible that changes in δ^15^N stem from an imbalance between protein synthesis and degradation within adrenal cancer cells. We also recognize that a general effect on protein metabolism and thus on serum δ^15^N due to cancer development is possible, in addition to a tumour-specific effect.

### 3.6. Hepatocarcinoma

The first δ^34^S values in patients with cancer were obtained using EA-IRMS analysis, and it has been found that both serum and erythrocytes are significantly ^34^S-depleted in patients with hepatocellular carcinoma (HCC) compared to controls [26]. It is plausible that this effect is due to change in the metabolism of S-containing amino acids, trans-sulphuration and methylation reactions which are intense in liver. In addition, glutathione (GSH) metabolism which plays important roles in cancer cells [55] could be involved, via oxidative stress response and the redox balance between extracellular cysteine and cysteine. For example, the formation of the disulphide bridge between cysteine molecules and thus in GSSG formation by GSH oxidation, fractionates against ^34^S [56,57]. In that context, the ^34^S-depletion in the serum of patients with HCC could come from a more oxidised status. Also, interestingly, it was found that δ^34^S values in patient with HCC patients correlated to the albumin level [58]. It is important to note δ^34^S values in serum of patients with BrCa or prostate cancer are not different from that in controls [58], showing that δ^34^S are probably specific to alterations in liver metabolism.

Presently, despite the rather small number of studies, it is clear that δ^15^N, δ^13^C and δ^34^S values have the potential to correlate to cancer development. Of course, since this effect is mostly caused by specific features of cancer cell metabolism, further studies with compound-specific analyses are required. This will be instrumental not only to better understand the metabolic origin of isotope signatures of cancer but also to find a reliable isotopic biomarker of cancer that is independent of nutrition (i.e., isotope composition of food intake) and population (country of residence) and therefore that do not require control samples for systematic comparison.

## 4. Isotope Fractionation in Metal Homeostasis

Isotope fractionations associated with four essential metals (Fe, Cu, Zn and Ca) as well as isotope compositions in various biological samples, including plants, animals and humans have been extensively reviewed in recent publications [27,59,60]. Here, we will very briefly explain principles of metal homeostasis and present latest studies dealing with metal isotope fractionation in human pathologies.

### 4.1. Copper Isotopes in Wilson and Menkes Diseases

**Figure 2:**
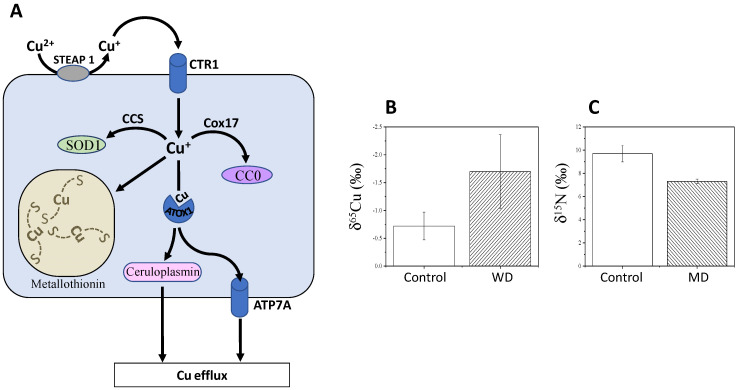
Isotope fractionations in Wilson and Menkes diseases. (**A**) Simplified Cu utilisation, including uptake via the transporter CTR1, intracellular redistribution to various molecules including storing in metallothionein, and efflux via the transporter ATP7A (also called Menkes ATPases) or A ceruloplasmin. When copper efflux capacity is by ATP7A is insufficient, metallothionein synthesis is induced and sequesters excess copper [27]. Mutations in ATP7B (not shown here) leads to Wilson disease (WD), which is characterized by an inability to excrete Cu into the bile and therefore hepatic Cu accumulation. (**B**) δ^65^Cu values in serum of WD patients [27] and (**C**) hair δ^15^N of baby patients with Menkes disease (MD) (*n* = 3) compared to control (*n* = 18) (unpublished data). See main text for further details on Cu homeostasis. Abbreviations: ATOX1, antioxidant 1 copper chaperone; CC0, cytochrome c oxidase; SOD, superoxide dismutase. In (**B**,**C**), delta values are significantly different between patients and controls (*p* < 0.05).

In principle, variations in ^65^Cu/^63^Cu ratios are due to the change in oxidation state (i.e., from Cu^2+^ to Cu^+^ or vice versa). In human and animals, diet cupric ions (Cu^2+^) are reduced to the cuprous form (Cu^+^) by the metalloreductase six-transmembrane epithelial antigen of prostate member 1 (STEAP1) and then absorbed by enterocytes via a specific transporter (CTR1) (Figure 2A). δ^65^Cu values in cells are lower than that in the diet, because Cu entering the cell is in its reduced form, which is ^65^Cu-depleted (isotope effect in reduction). Cu is then transported from the intestine to the liver and used to synthesise Cu-containing proteins, such as copper chaperone for superoxide dismutase (CCS, which delivers Cu to superoxide dismutase, SOD1), cytochrome c copper chaperone (Cox17) (which delivers copper to cytochrome c oxidase, CCO), ATP7A/7B (copper transporters) and ceruloplasmin (major copper-carrying protein in blood). Cu transfers occur through oxidation reactions, inducing an increase in δ^65^Cu in SOD1, CCO and ceruloplasmin compared to source Cu. In addition to the change in oxidation state, forming specific chemical bonds also fractionates between Cu isotopes. For example, forming a Cu-S bond discriminates against ^65^Cu, while forming a Cu-O or Cu-N bond discriminates in favour of ^65^Cu. This explains why the liver, which accumulates the Cu- and S-containing metal-binding protein metallothionein, has a low δ^65^Cu value compared to other organs in sheep [61]. Conversely, δ^65^Cu values are high in kidney since Cu binds to oxalate and carbonate via Cu-O bonds [61]. Also, δ^65^Cu values are elevated in bone, likely because of the formation of Cu-O bonds in hydroxyapatite, the main mineral in bone.

Because of its involvement in SOD1 catalysis, Cu is involved in the mitigation of reactive oxygen species (ROS), which are thus influenced by intestinal absorption and bile excretion of Cu [27]. Alterations in Cu homeostasis can cause serious diseases, such as Wilson and Menkes diseases [27]. The Menkes disease (MD) involves a mutation in ATP7A and leads to copper deficiency and thereby strong neurodegenerative disorders. Mutations in ATP7B leads to Wilson disease (WD), which is characterized by an inability to excrete Cu into the bile and therefore hepatic Cu accumulation. WD is characterized by cerebral and cerebellar degeneration, failure to thrive, coarse hair and connective tissue abnormalities [62]. Unlike WD, no extensive Cu isotope data has been reported for patients with MD. However, hair δ^15^N has been shown to increase in babies with MD (Figure 2B) while no change was found in δ^13^C values. Low δ^65^Cu values are observed in the serum of patients with WD, compared to healthy subjects (Figure 2C). 

This effect probably comes from the decrease in the level of blood ceruloplasmin, which is ^65^Cu-enriched. Lamboux and co-workers [63] reported recently that healthy subjects and naïve (non-treated) patients with WD had undistinguishable δ^65^Cu, and treated patients had the same δ^65^Cu values regardless of treatment type and duration. However, the variation in serum δ^65^Cu was negatively correlated with the degree of liver fibrosis. This suggests that the inability for Cu recirculation from the liver is at the origin of the ^65^Cu-depletion in general circulation, regardless of the genetic background and treatment. Also, as the cuprous form (^65^Cu-depleted) accumulates in the liver, the production of free radicals increases and participates in triggering WD symptoms [63]. These results suggest that δ^65^Cu is not a good biomarker of ATP7B mutation but rather, has some potential as a prognostic biomarker for evaluating the progression of liver fibrosis in WD.

Interestingly, changes in δ^65^Cu can also be observed in diseases other than WD and MD. For example, in patients with ovarian cancer, δ^65^Cu values in plasma are lower than in healthy controls [28]. δ^65^Cu values in ovary tumour tissues are higher than in adjacent healthy tissues [28], simply suggesting a mass-balance effect whereby the increased Cu influx in tumours forms ^65^Cu-enriched copper lactate [26] and depletes healthy ovary tissues and blood in ^65^Cu.

### 4.2. Multiple Metal Isotopes in Other Pathologies

Recent studies used the combination of Cu, Fe and Zn isotopes in blood to assess possible homeostasis alterations after bariatric surgery and during the follow-up of haematological malignancy (HM) in patients [29,30].

Both serum and whole blood had lower δ^65^Cu values after bariatric surgery, reaching statistical significance at 6 months post-surgery [30]. Although micro-elements supplementation of patients is unknown is that study, the current hypothesis is that duodenal Cu absorption and mineral bioavailability (and the amount of digestive juice in the stomach) are limited due to the gastric bypass. By contrast, serum ^66^Zn was slightly higher 6 months post-surgery than pre-surgery, but the difference did not reach statistical significance and furthermore, this enrichment in ^66^Zn was not observed in whole blood. Still, the difference in δ^66^Zn value between serum and whole blood (expressed as Δ^66^Zn) became gradually larger over post-operative time. This effect was not observed for Δ^65^Cu and Δ^56^Fe. Presumably, the change in Δ^66^Zn might reflect a disruption in Zn homeostasis, Zn status or Zn absorption (and competition with Cu absorption) in patients with bariatric surgery. When the Zn supply is low, Zn is remobilised from stored Zn to fulfil metabolic requirements. Intracellular Zn is bound to metallothionein via a Zn–S bonds (involving cysteine residues) and is released when blood Zn level is low [27]. As the Zn–S bond formation discriminates against ^66^Zn, ^66^Zn isotope would be preferentially released from intracellular metallothionein to serum, causing a general change in δ^66^Zn of circulating Zn.

Like Cu isotopes, Fe isotopes are extremely useful to follow alterations in the different steps of Fe homeostasis [27]. Patients suffering from severe diabetes and Fe deficiency anaemia show high δ^56^Fe values in whole blood. This effect is due to changes in the nutritional status of Fe, redox reactions during Fe absorption in the intestine, Fe storage in the liver, or the synthesis of Fe-containing proteins (typically haemoglobin or myoglobin). Fe^3+^ from diet is reduced to Fe^2+^ before being absorbed by the intestine, causing a low δ^56^Fe in intestine. The δ^56^Fe value is higher in the liver, where Fe is stored into ferritin in its Fe^3+^ form (naturally enriched compared to Fe^2+^). Reciprocally, the Fe^3+^ ion of transferrin (Tf) is reduced to Fe^2+^ in haemoglobin and myoglobin, leading to a low δ^56^Fe value in red blood cells and muscles [27,59]. Unlike Cu and Fe, Zn is not a redox sensitive element. Therefore, metabolic isotope fractionations with Zn isotopes are numerically smaller than those found with Cu and Fe. However, large variations in Zn isotopes are observed between plants, animals and humans [27]. They are attributable to the high binding energy of Zn with ligand molecules and thus effects of Zn adsorption and coordination at the root surface in plants, which are very different to biochemistry of animal Zn absorption.

In humans, Ca is the most abundant metal element in the body where it plays an important role in neurotransmission, muscle contraction, and regulation of volumes of biological fluids. Originally, it was believed that there was a big Ca isotope fractionation during bone formation (apatite deposition). Nevertheless, it was then observed that the δ^44^Ca value in blood and bone was similar. The largest difference was found between urine and blood, simply because of Ca isotope fractionation during kidney reabsorption of Ca from primary urine (from glomerular filtrate) into blood. Serum δ^44^Ca in patients with multiple myeloma (MM) is lower than in healthy controls [31]. In fact, MM is characterized by osteolytic lesions (bone mass loss) due to a relative increase in osteoclastic activity, liberating Ca^2+^ and causing bone resorption. Primary urine has thus a higher load in Ca with relatively low δ^44^Ca (compared to normal primary urine), which is then reabsorbed into blood, explaining a low δ^44^Ca in serum of patients with MM [31]. Tanaka et al. (2017) [32] reported that serum and bone from rats with chronic kidney disease or diabetes had lower δ^44^Ca values than in controls. δ^44^Ca values were correlated to bone mineral density, suggesting a link with bone resorption and formation like in MM. The Ca isotopic analysis of both urine and blood thereby offers a non-invasive method for investigating bone-related diseases, perhaps including early detection and assessment of treatment efficacy.

## 5. Isotopes in Skeletal Pathologies

Examples described in above sections are case studies of pathologies studied with living subjects. Stable isotopes can also provide information on past physiological conditions from archaeological remains. For example, skeletal stress indicators are used in bioarchaeology to inform on past palaeopathologies such as scurvy, osteoporosis, vitamin D deficiency (rickets and osteomalacia), endocrine disorders (e.g., acromegaly), haematological disorders (e.g., anaemia) and infectious diseases (e.g., tuberculosis, treponemal diseases, and leprosy as indicated by periostitis and osteomyelitis) [34].

### 5.1. Oxygen Isotopes in Sickle-Cell Anaemia

Only two studies [33,34] have demonstrated that enamel apatite δ^18^O values may provide a biological markers for anaemia in skeleton fragments. The first study showed a relationship between δ^18^O of bone apatite and mice expressing human Sickle-Cell anaemia haemoglobin HbS [64]. Bone apatite δ^18^O of sick mice were significantly lower than those of healthy mice, and the sickest mice exhibited the lowest δ^18^O [64]. These results were confirmed in the enamel apatite samples of suspected anaemics, which had significantly lower δ^18^O values relative to their lesion-free counterparts [33].

It is believed that the rate of cellular metabolism and the rate of total respiration is reflected by stable oxygen isotopes in dental enamel apatite (Ca_3_(PO_4_)_2_), via (i) oxygen (O_2_) reduction to metabolic water (H_2_O) during respiratory biochemistry and then (ii) isotopic exchange between tissue water and phosphate oxygen atoms. In effect, organism respiration fractionates against ^18^O and this comes from two main phenomena. First, there is an oxygen isotope fractionation as inhaled dioxygen (O_2_) diffuses through alveolar membranes to pulmonary capillaries. During this process, the rate of ^16^O_2_ diffusion is about 3‰ larger than that of ^16^O-^18^O and furthermore, there is an equilibrium isotope effect during O_2_ binding to haemoglobin, leading to an ^18^O-depletion in oxygen carried by the vascular system [33]. Second, a fractionation occurs once O_2_ is consumed by cellular respiration and converted to water (H_2_O), since mitochondria utilise ^16^O_2_ about 13‰ faster than ^16^O-^18^O [33]. Given the pathophysiology of anaemias, three potential causes can explain low δ^18^O values. First, oxygen fixation is slow due to lower functional haemoglobin and/or fewer overall erythrocytes and thus the fractionation is maximal (kinetic effect). Second, O_2_ binding by HbS may fractionate more against ^18^O than normal haemoglobin. This explanation is less likely since low δ^18^O are found also in anaemics without sickle cells disease. Third, metabolic adaptation of cellular metabolism is so that mitochondria do not have the same isotope fractionation during metabolic water generation by respiration. There is presently no definitive evidence to select one of these three hypotheses.

### 5.2. Nitrogen and Carbon Isotopes in Collagen

It is also known that anaemia inhibits bone matrix formation, increases bone resorption, and adversely affects collagen maturation, thereby increasing risks of osteopenia and osteoporosis. Interestingly, bone collagen of females with osteopenia has been found to be significantly enriched in ^15^N [33]. The authors suggested that the enrichment may be a result of underlying differences in urea excretion and/or renal function [33], both of which being expected to be altered in anaemia-induced osteopenia and osteoporosis. As such, bone collagen of individuals with severe manifestations of anaemia could be anticipated to be ^15^N-enriched (and perhaps ^13^C-enriched) relative to healthy individuals. However, in another study there is no significant variation in bone collagen δ^13^C or δ^15^N values between anaemic and lesion-free juveniles or adults [33]. This may be due to a number of factors, including age, the small sample size and the sampling strategy employed (possible bias in life habits and co-pathologies). It may also indicate that the pathophysiology of anaemia must be chronic to alter significantly the isotope composition in bones. More research, with a more refined sampling strategy and larger sample sizes, as well as isotopic measurements in each individual amino acid and other skeletal tissues, such as dentin and bone bicarbonate, could be of interest for future research.

δ^15^N and δ^13^C can be used to trace traumatic events and aggravation of lesions in progressive diseases. Delta values were measured in several skeletons to assess whether different stages of bone-related disease may affect bone collagen isotope composition [65]. This study used the cortical bone layer, which retains evidence of lesions caused by disease or healed fractures and compared visible lesions with cortical bone from the same individual without lesions. Long bones with active lesions were found to have a significantly higher δ^15^N suggesting increased protein metabolism or collagen deposition. Fracture calluses showed the largest range for both δ^15^N and δ^13^C, related to different healing stages. But surprisingly, unlike long bones, there were no significant differences in delta values between non-lesion and lesion sites in ribs. In fact, in ribs it is more difficult to separate abnormal new bone from underlying cortical bone, because lesions and non-lesion sites are visually similar [65].

## 6. Conclusions and Perspectives

In this mini-review, we have made apparent the fact that many human diseases have consequences on the natural abundance of stable isotopes, not only in affected tissues themselves but also in more accessible sample types, such as plasma, urine or hairs. This explains why many research projects across the world deal with the utilisation of delta values as potential diagnostic and prognostic tools. However, it should be recognised that there are presently two conundrums to solve in order to envisage clinical implementation. First, better (more sensitive and selective) biomarkers are likely to be represented by delta values in specific metabolites rather than total organic matter (raw samples). This implies sample preparation and analysis with compound-specific techniques (such as GC-c-IRMS) which are often not suitable to medical use due to sample size and the time required for analysis. Second, there is a lack of knowledge of specific metabolic causes of isotope fractionations in key processes and thus, precise mechanisms at the origin of the isotopic signature of diseases are incompletely understood. To fill this gap of knowledge, having more data on both metabolic fluxes and enzymatic isotope effects is necessary. While the investigation of metabolic flux patterns (fluxomics) is blooming, there is little work made on the measurement of isotope effects. The present paper is a call for more basic research in this area, so as to facilitate the discovery of new diagnostic biomarkers based on stable isotopes. In effect, once characteristic isotope fractionations associated with diseases will be determined, this will open avenues for patient management. Early cancer detection and diagnosis is presently one of the most exciting research area (Section 3 above), with promising and reproducible isotopic patterns found in tissues and cultured cells. Refined isotopic analyses (including compound-specific data) in the future will certainly be instrumental to help monitoring results of cancer therapies or provide a biomarker for prognostic stratification.

## Figures and Tables

**Table 1:** Summary of documented examples of pathologies where isotopes at natural abundance could be used for potential diagnostics. Aa, amino acid; ND, not determined. The term “metabolic mechanism” refers to the major pathways explaining the change in isotope abundance.

Disease	Metabolic Mechanism	Isotopic Marker	Matrix	Ref.
Nervous anorexia, nutritional stress	Aa metabolism	^13^C, ^15^N	Hair	[10,11]
Syphilis	Aa metabolism	^13^C, ^15^N	Collagen	[12]
Chronic malnutrition and potential growth retardation (stunted children)	Aa metabolism	^13^C, ^15^N	Hair	[13]
Patients with metabolic syndrome	Glycaemia Aa metabolism	^13^C, ^15^N	Hair	[14]
Diabetic patients	Sugar metabolism	^13^C, ^15^N	Hair	[15,16,17,18]
Cirrhotic patients	Aa metabolism	^13^C, ^15^N	Hair, bulk protein	[19]
Breast cancer	Urea cycle, glycolysis, lipid synthesis, anaplerosis	^13^C, ^15^N	Tissue biopsies cultured cells	[20,21]
Oral squamous cell carcinomas	ND	^13^C, ^15^N	Tissue biopsies	[22]
Ganglioneuroma (benign tumours), neuroblastoma and nephroblastoma Wilm’s tumours	Aa metabolism	^13^C, ^15^N	Tissue biopsies	[23,24]
Rhabdomyosarcoma	ND	^13^C, ^15^N	Tissue biopsies	[25]
Adrenal gland cancers	Aa metabolism Glycolysis	^13^C, ^15^N	Serum	*Unpublished data*
Hepatocarcinoma	Glutathione metabolism,	^34^S	Serum and erythrocytes	[26]
Wilson disease	Cu metabolism	^65^Cu	Serum	[27]
Menkes disease	Cu and Aa metabolism	^15^N	Hair	*Unpublished data*
Ovarian cancer	Cu metabolism	^65^Cu	Serum	[28]
Homeostasis alterations after bariatric surgery	Zn homeostasis	^66^Zn	Serum and Whole blood	[29]
Hematological malignancy	Metal homeostasis	^65^Cu, ^66^Zn	serum	[30]
Anaemia	Fe deficiency	^56^Fe	Whole blood	[27]
Multiple myeloma	Bone formation (apatite deposition)	^44^Ca	Serum and urine	[31]
Chronic kidney disease or diabetes	Bone formation (apatite deposition)	^44^Ca	Serum	[32]
Anaemia in skeleton fragments	Respiratory biochemistry	^18^O	Bone and enamel apatite	[33]
Osteopenia and osteoporosis in female skeleton	Urea excretion and/or renal function	^15^N	Bone collagen	[34]
Cealiac disease in skeleton	Aa metabolism	^13^C, ^15^N	Bone collagen	[35]

## Appendix A

Box A1 Isotope Composition and fractionation.

***δ-value or isotope composition*** (usually expressed in ‰): abundance of the heavy isotope with respect to that of the light isotope, as compared to the international standard material. It is denoted as δ^13^C for carbon, δ^15^N for nitrogen, etc.
***Fractionation*** (Δ): isotopic difference (usually expressed in ‰) between the substrate and the product of a reaction. It is given by the deviation of the isotope effect (*α*) from 1, as Δ = *α* − 1. It can be shown that it can be calculated from δ-values in substrate and product as: Δ = (δ_substrate_ − δ_product_)/(δ_product_ + 1).
***Isotope effect*** (*α*): ratio of rate constants (*k*_light_/*k*_heavy_) or equilibrium constants (*K*_light_/*K*_heavy_) of the isotopologues of interest. For enzymatic reaction, it is the ratio of catalytic efficiency *V/K*: *α* = (*V/K*)_light_/(*V/K*)_heavy_.
***Isotopologue***: isotopic analogue of a molecule, where one atom has been replaced by its isotope. For example, ^13^C^16^O_2_ is the ^13^C-isotopologue of ^12^C^16^O_2_. It must not be confused with “isotopomers”, which refer to isotopic isomers (for example, ^13^CH_3_–^12^CH_2_OH and ^12^CH_3_–^13^CH_2_OH are two isotopomers of ethanol).
***Isotope ratio mass spectrometer*** (IRMS): mass spectrometer based on a magnetic sector with (usually) fixed collectors (Faraday cups) adapted to quantify precisely the abundance of isotopic species of CO_2_ (^13^C analysis), N_2_ (^15^N analysis), CO (^18^O analysis), H_2_ (^2^H analysis) or SO_2_ (^34^S analysis). The historical origin and technical principles of IRMS are reviewed in [66].
***Quantitative reaction***: chemical reaction that consumes all of the substrate molecules, thereby preventing any isotope fractionation. In non-quantitative reactions, substrate molecules left behind may have a δ-value different from the initial value because the reaction selects for an isotopic species (isotope effect).

Box A2 Isotopic analyses. The natural abundance of stable heavy isotopes (^13^C, ^15^N, ^34^S, ^18^O, ^2^H, etc.) is relatively low for some elements (about 0.01% for ^2^H for example, while other are abundant, e.g., about 4.3% for ^34^S) and variations caused by metabolic fractionations are small (a few ‰). Therefore, techniques used to measure isotope natural abundance and its modification in pathologies utilise specific devices with high sensitivity and low measurement errors. The instrument used depends on elements and the type of sample (raw material, extract, etc.).

- *Analysis of raw material (total organic matter)*: typically, raw samples are analysed by elemental analysis (EA) coupled to isotope ratio mass spectrometry (IRMS). In the EA, samples are combusted to CO_2_ and N_2_ (^13^C and ^15^N analysis) or pyrolised to CO and H_2_ (^18^O and ^2^H analysis). The IRMS measures the mass ratio (45/44 for CO_2_, 29/28 for N_2_, etc.) that are then converted to isotope ratios (^13^C/^12^C, ^14^N/^15^N, etc.). IRMS measurements requires comparison with a reference gas of known δ value. Alternatively, the δ value of the reference gas can be determined by comparison of a certified standard sample of known δ value from the international agency for atomic energy (IAEA, Vienna, Austria). EA-IRMS analyses are adapted to raw samples (lyophilised biopsies or exeresis samples), or pre-purified tissue components (e.g., precipitated proteins, extracted lipids).
- *Compound-specific analyses*: the isotope composition of specific, targeted metabolites can be determined via three methods: (*i*) pre-purification with preparative chromatography followed by EA-IRMS; (*ii*) liquid chromatography coupled to IRMS via a chemical oxidation interface (LC-co-IRMS); and (*iii*) gas chromatography coupled to IRMS via a combustion interface (GC-c-IRMS). Method (*i*) has been used extensively in the 90s in plant biology when LC-co-IRMS and GC-c-IRMS were not available. However, this method requires large amounts of material incompatible with small medical samples. Method (*ii*) is currently limited to carbon isotopes and metabolites that can be resolved using water as an eluent in the LC system. Method (*iii*) is widely used, and there is now an enormous associated literature, reviewed in (Tea and Tcherkez [67]).
- *Gas analyses*: gas analysis mostly concerns CO_2_ produced by respiration and collected in exhaled breath air. There are presently two main techniques to determine the δ^13^C value in respired CO_2_: (*i*) laser-based techniques, and (*ii*) IRMS-based techniques. Laser-based techniques take advantage of the difference in absorptance between ^12^CO_2_ and ^13^CO_2_ to compute the ^13^C/^12^C ratio of a gas sample. This is a rather simple and instantaneous method that is now implemented routinely for the detection of stomachal ulcers to monitor ^13^CO_2_ production from ^13^C-urea. To perform precise measurements at natural abundance, however, laser-based systems require time-consuming calibration curves not only for CO_2_ mole fraction but also for δ^13^C values. This implies the need of gas cylinders at different CO_2_ concentration and δ values, and thus IRMS measurements for cross-validation. IRMS-based techniques simply use a GC-IRMS coupling whereby air constituents are separated by GC and the CO_2_ peak is injected into the IRMS. Analyses are slower than with laser systems but provide a precise value with the same requirements as other IRMS measurements.
- *Heavy atom isotope analyses*: since most IRMS systems analyse simple gases (CO_2_, N_2_, H_2_, SO_2_, CO), they cannot convey metal analyses. To do so, multi-collectors mass spectrometers are required. In such systems, the sample of interest is broken down by inductively coupled plasma (ICP) and isotope analysis is performed at the atomic level (instead of gases). The clear advantage of this type of instrument is its versatility, because many elements can be analysed just by changing source parameters, not only metals (Mg, Fe, Cu, etc.) but also macroelements (such as S).
- *Other techniques*: there is now a considerable interest in techniques that can provide information at the intramolecular level, and not solely a molecular average δ value. In fact, metabolic pathways are so that metabolites are fragmented and assembled and therefore strong differences in δ value are anticipated between atom positions within a metabolite. In plant biology, this topic is currently an intense area of research. However, current methods use nuclear magnetic resonance (NMR) which requires quite large amounts of material incompatible with medical samples. Alternative techniques such as Orbitrap^®^-based analyses are currently under consideration but not applicable to complex mixtures or small samples.
